# Benefits of intensive insulin therapy on neuromuscular complications in routine daily critical care practice: a retrospective study

**DOI:** 10.1186/cc7694

**Published:** 2009-01-24

**Authors:** Greet Hermans, Maarten Schrooten, Philip Van Damme, Noor Berends, Bernard Bouckaert, Wouter De Vooght, Wim Robberecht, Greet Van den Berghe

**Affiliations:** 1 Medical Intensive Care Unit, Department of Internal Medicine, University Hospitals Leuven, Catholic University Leuven, Herestraat 49, B-3000 Leuven, Belgium; 2 Department of Neurology, University Hospitals Leuven, Catholic University Leuven, Herestraat 49, B-3000 Leuven, Belgium; 3 Laboratory for Neurobiology, Department of Experimental Neurology, Flemish Institute for Biotechnology, Catholic University Leuven, Herestraat 49, B-3000 Leuven, Belgium; 4 Department of Intensive Care Medicine, University Hospitals Leuven, Catholic University Leuven, Herestraat 49, B-3000 Leuven, Belgium

## Abstract

**Introduction:**

Intensive insulin therapy (IIT) reduced the incidence of critical illness polyneuropathy and/or myopathy (CIP/CIM) and the need for prolonged mechanical ventilation (MV ≥ 14 days) in two randomised controlled trials (RCTs) on the effect of IIT in a surgical intensive care unit (SICU) and medical intensive care unit (MICU). In the present study, we investigated whether these effects are also present in daily clinical practice when IIT is implemented outside of a study protocol.

**Methods:**

We retrospectively studied electrophysiological data from patients in the SICU and MICU, performed because of clinical weakness and/or weaning failure, before and after routine implementation of IIT. CIP/CIM was diagnosed by abundant spontaneous electrical activity on electromyography. Baseline and outcome variables were compared using Student's t-test, Chi-squared or Mann-Whitney U-test when appropriate. The effect of implementing IIT on CIP/CIM and prolonged MV was assessed using univariate analysis and multivariate logistic regression analysis (MVLR), correcting for baseline and ICU risk factors.

**Results:**

IIT significantly lowered mean (± standard deviation) blood glucose levels (from 144 ± 20 to 107 ± 10 mg/dl, p < 0.0001) and significantly reduced the diagnosis of CIP/CIM in the screened long-stay patients (125/168 (74.4%) to 220/452 (48.7%), p < 0.0001). MVLR identified implementing IIT as an independent protective factor (p < 0.0001, odds ratio (OR): 0.25 (95% confidence interval (CI): 0.14 to 0.43)). MVLR confirmed the independent protective effect of IIT on prolonged MV (p = 0.002, OR:0.40 (95% CI: 0.22–0.72)). This effect was statistically only partially explained by the reduction in CIP/CIM.

**Conclusions:**

Implementing IIT in routine daily practice in critically ill patients evoked a similar beneficial effect on neuromuscular function as that observed in two RCTs. IIT significantly improved glycaemic control and significantly and independently reduced the electrophysiological incidence of CIP/CIM. This reduction partially explained the beneficial effect of IIT on prolonged MV.

## Introduction

Critical illness polyneuropathy (CIP) is an acute and primary axonal motor and sensory neuropathy that typically occurs in critically ill patients as a complication of their illness and possibly its therapy [1]. The signs and symptoms are not always easily distinguished from critical illness myopathy (CIM), which is a primary muscle disease that may occur in the same setting [2]. Both CIP and CIM also frequently occur simultaneously [3-5], and therefore, from a clinical point of view, both are often grouped together as critical illness polyneuropathy and/or myopathy (CIP/CIM). They result in limb and respiratory muscle weakness, causing difficulty in weaning from the ventilator and impaired rehabilitation [6-9]. CIP/CIM is therefore associated with prolonged intensive care unit (ICU) and hospital stay and increased mortality rates [6,8,10]. Differentiation between both conditions is possible in some patients using nerve conduction studies (NCS) and needle electromyography (EMG). However, the differential diagnosis between CIP and CIM on routine electrophysiological examination is frequently hampered by tissue oedema, interfering with correct sensory nerve action potential (SNAP) assessment, and the inability to voluntarily contract muscles, interfering with correct motor unit potential analysis.

The pathophysiology of CIP/CIM is very complex and many factors and mechanisms, such as electrical, microvascular, metabolic alterations, bioenergetic failure and altered Ca^2+ ^homeostasis, have been suggested to explain the observed changes in the neural and muscular system [11]. Also, different risk factors for CIP/CIM development have been identified in several prospective studies. These include systemic inflammatory response syndrome (SIRS) and multiple organ failure (MOF), in which severity of illness [4,12] and duration of organ dysfunction [13] seem to be crucial. Other risk factors identified include hyperglycaemia [14,15], vasopressor and catecholamine support [15], neuromuscular blocking agents [9], corticosteroids [13], female sex [13], hypoalbuminaemia [14], parenteral nutrition [10], hyperosmolarity [10], renal replacement therapy [10], duration of ICU stay [14,15] and central neurological failure [10]. Not all risk factors have been consistently identified and many remain controversial.

Until recently, prevention of CIP/CIM was solely based on minimising the effects of these identified risk factors. However, in two randomised controlled trials (RCTs) in a surgical ICU (SICU) [15] and medical ICU (MICU) [9], our group has demonstrated that intensive insulin therapy (IIT) aimed at blood glucose levels between 80 and 110 mg/dl, significantly reduced the electrophysiological incidence of CIP/CIM and also the need for prolonged mechanical ventilation (MV) in the subpopulation of patients with an ICU stay of at least one week. Indeed, hyperglycaemia had been previously identified to be associated with CIP/CIM development. Potential mechanisms are impairment of the microcirculation in the nerve and mitochondrial dysfunction because of an increased generation/deficient scavenging of reactive oxygen species. In addition, insulin itself may have some benefits by affecting the balance between anabolic and catabolic hormones.

As the beneficial effect of IIT has been observed in the setting of RCTs, we further studied whether the implementation of IIT in routine daily ICU practice and outside a study protocol would result in similar beneficial effects on neuromuscular electrophysiology.

## Materials and methods

We retrospectively evaluated all electronically available electrophysiological data derived from NCS/EMG in patients in the SICU and MICU before and after implementation of IIT in routine clinical practice. For this purpose, only NCS/EMG performed because the treating physician noticed a clinical problem of weakness and/or weaning failure were selected and therefore the study sample comprised only a subset of the long-stay ICU population. We diagnosed CIP/CIM solely based on the presence of abundant spontaneous electrical activity in the form of positive sharp waves and/or fibrillation potentials. Excluded from the study were patients with an NCS/EMGs suggesting diagnoses other than CIP/CIM, patients under the age of 18 and those with technically inconclusive examinations, as well as all data of patients included in the previous RCTs.

To explore the effects of IIT on CIP versus CIM, we compared patients in whom reliable contraction patterns could be obtained, allowing identification of primarily myopathic pathology. However, this can not be achieved in all patients. Because reduction in amplitude of the SNAPs are suggestive of CIP (and not encountered in pure CIM without accompanying CIP) we also studied the SNAPs before and after implementation of IIT. Finally, the need for prolonged MV, defined as MV for at least 14 days, as in the previous trials [9,15], was recorded. This study was approved by the local ethics committee. As it concerned retrospective analysis of data obtained during usual clinical practice, local regulations do not require informed consent to be obtained.

### Statistics

Data were analysed using Statview 5.0 (SAS Institute, Inc., Cary, NC). Baseline and outcome variables are presented as mean ± standard deviation if normally distributed, and median and interquartile range if skewed. Data were compared using Student's t-test, Chi-squared test or Mann-Whitney U test when appropriate. The effect of implementing IIT in daily practice on CIP/CIM and prolonged mechanical ventilation was assessed using univariate analysis. Next, also multivariate logistic regression analysis (MVLR) was used to evaluate the effect of IIT on CIP/CIM and prolonged MV. We included in the model, all baseline factors and risk factors that occurred during ICU stay that either showed an imbalance between the groups before and after implementation of IIT (p ≤ 0.1) or showed at least a trend in the univariate analysis (p ≤ 0.1) on CIP/CIM, respectively prolonged mechanical ventilation.

## Results

### Patient characteristics

**Figure 1:**
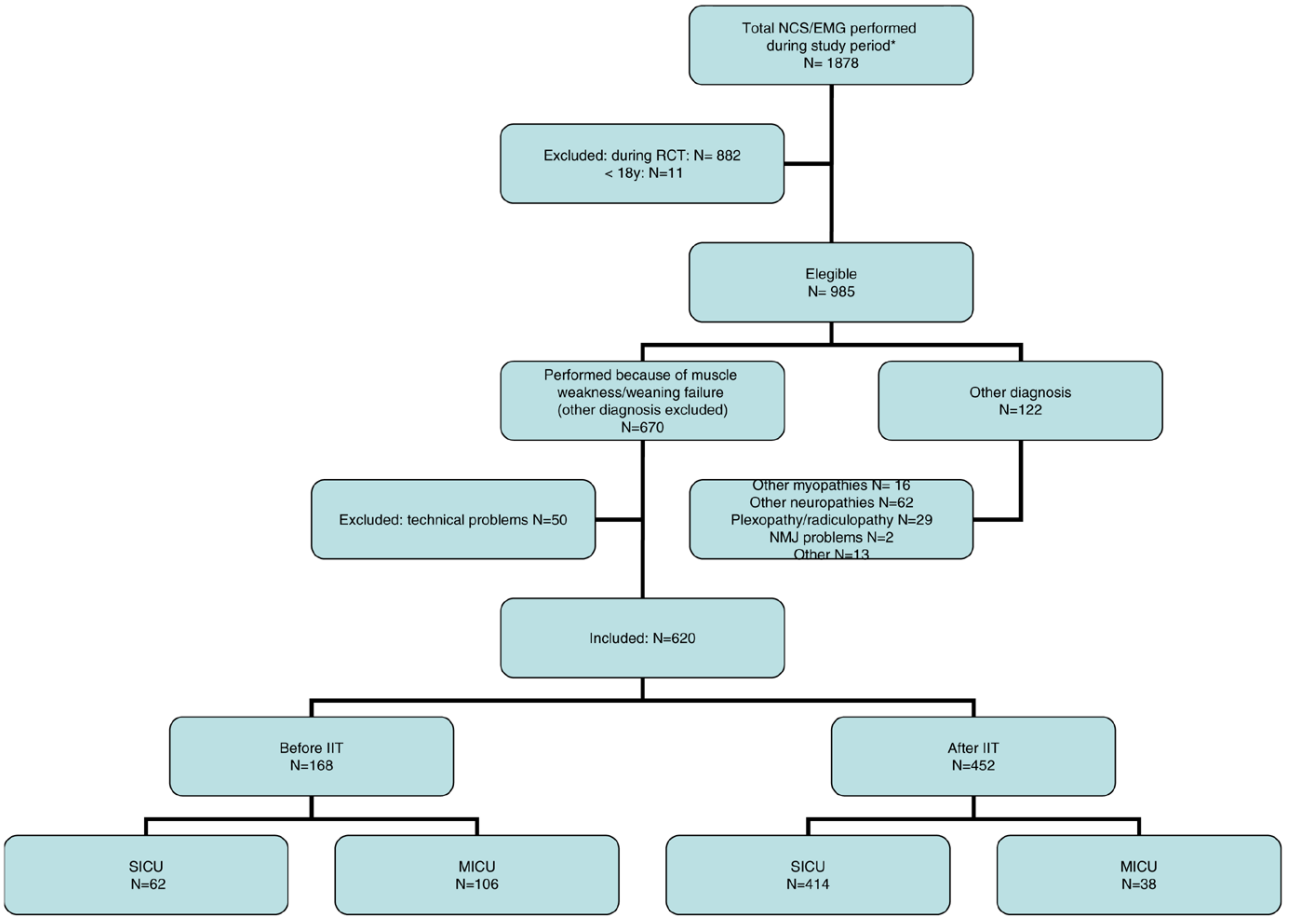
CONSORT diagram of the study. IIT = intensive insulin therapy; MICU = medical intensive care unit; SICU = surgical intensive care unit.

**Figure 2:**
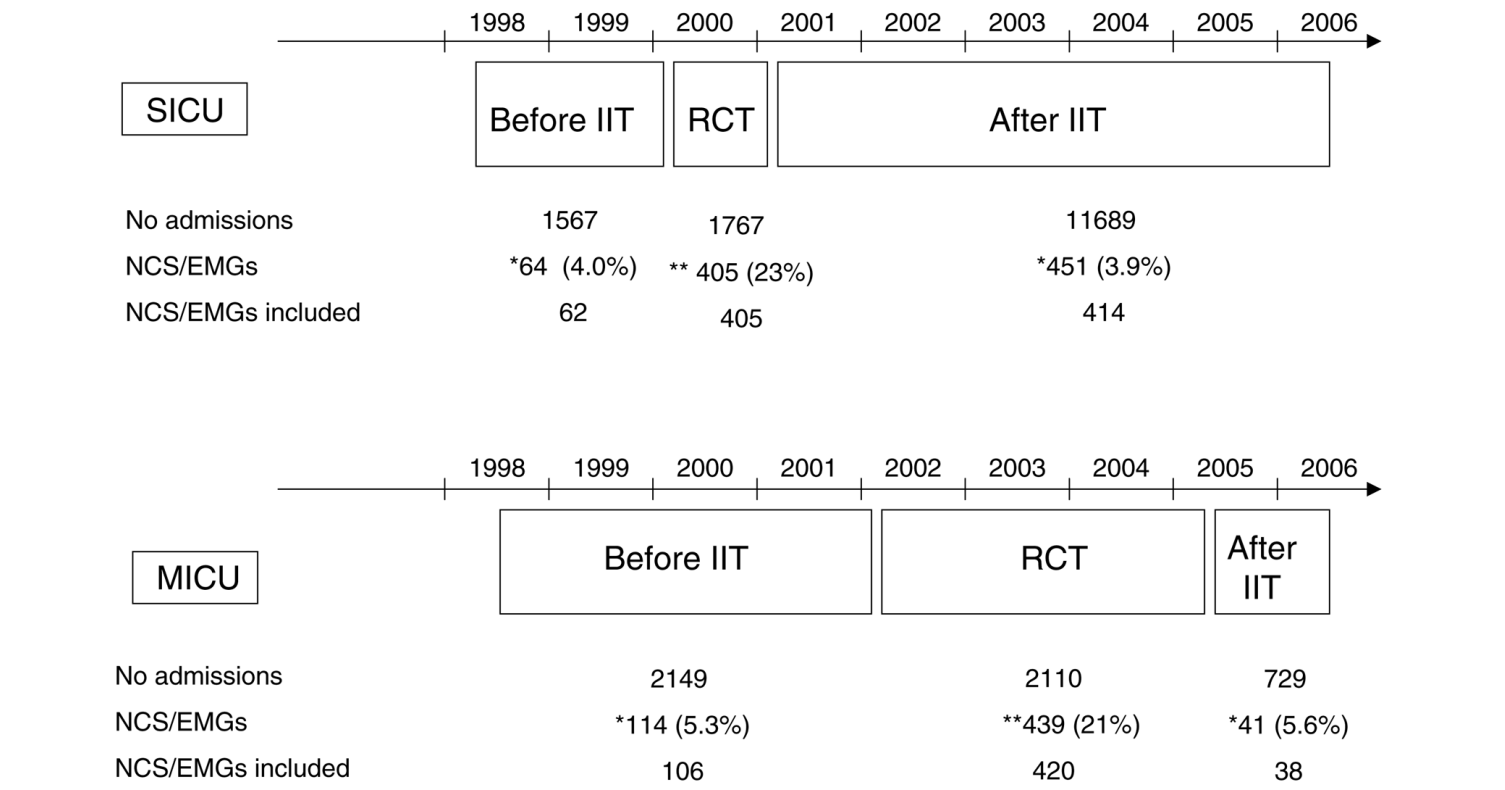
Chronological order of the study. Data were collected from patients in both intensive care units (ICUs) before the randomised controlled trials (RCTs). After the trials intensive insulin treatment was implemented in both ICUs. EMG = needle electromyography; IIT = intensive insulin therapy; MICU = medical intensive care unit; NCS = nerve conduction studies; SICU = surgical intensive care unit.

After excluding other diagnoses, NCS/EMGs of a total of 620 patients performed because of weakness and/or weaning failure were included in the analysis (Figures 1 and 2). This included 168 patients in the ICU before and 452 after the implementation of IIT. The proportion of patients receiving NCS/EMGs before and after the RCTs and the implementation of IIT in daily practice was not different (MICU before: 5.3%, after: 5.6%, SICU before: 4.0% after: 3.9%). Baseline characteristics of these patients are shown in Table 1.

**Table 1:** Baseline characteristics of the studied sample of long-stay patients

	**Total population n = 620**			**Surgical intensive care unit n = 476**			**Medical intensive care unit n = 144**		
			
	Before IIT n = 168	After IIT n = 452	p-value	Before IIT n = 62	After IIT n = 414	p-value	Before IIT n = 106	After IIT n = 38	p-value
Male/female sex, n (%)	105/168 (62.5)	305/452 (67.5)	0.2	41/62 (66.1)	285/414 (68.8)	0.7	64/106 (60.4)	20/38 (52.6)	0.4
Age, years (mean ± SD)	61 ± 15	62 ± 14	0.4	64 ± 13	63 ± 14	0.6	60 ± 15	61 ± 17	0.9
ICU type/MICU total n (%)	106/168 (63.1)	38/452 (8.4)	**< 0.0001**						
Diagnostic group, total n (%) of the category			**< 0.0001**			0.1			
Abdominal/gastro- intestinal/liver	19/71 (26.8)	52/71 (73.2)		6/55 (10.9)	49/55 (89.1)		13/16 (81.3)	3/16 (18.7)	
Cardiovascular	24/171 (14.0)	147/171 (86.0)		21/167 (12.6)	146/167 (87.4)		3/4 (75.0)	1/4 (25.0)	
Cerebral/neurological	6/60 (10.0)	54/60 (90.0)		2/52 (3.8)	50/52 (96.2)		4/8 (50.0)	4/8(50.0)	
Haematological/oncol ogy/transplant	3/31 (9.7)	28/31 (90.3)		2/29 (6.9)	27/29 (93.1)		1/2 (50.0)	1/2 (50.0)	
Other	32/73 (43.8)	41/73 (56.2)		10/43 (23.3)	33/43 (76.7)		22/30 (73.3)	8/30 (26.7)	
Polytrauma	6/37 (16.2)	31/37 (83.8)		6/37 (16.2)	31/37 (83.8)		0/0	0/0	
Respiratory/thoracic	61/136 (44.9)	75/136 (55.1)		8/64 (12.5)	56/64 (87.5)		53/72 (73.6)	19/72 (26.4)	
History of diabetes, total n (%)			0.2			0.9			0.07
Insulin treated	11/151 (7.3)	26/420 (6.2)		3/55 (5.5)	23/384 (6.0)		8/96(8.3)	3/36 (8.3)	
Oral antidiabetic treatment and/or diet	16/151 (10.6)	26/420 (6.2)		3/55 (5.5)	26/384 (6.8)		13/96 (13.5)	0/36 (0)	
Baseline APACHE II, (mean ± SD)	19.0 ± 8.3	16.2 ± 7.1	**< 0.0001**	14.6 ± 6.7	15.7 ± 6.9	0.3	21.7 ± 8.1	21.5 ± 7.5	0.9
On admission blood glucose, mg/dl median (IQR)	157 (126 to 202)	134 (107 to 172)	**< 0.0001**	163 (126 to 199)	135 (109 to 173)	**0.007**	151 (126 to 202)	124 (96 to 156)	**0.008**
On admission mechanical ventilation, total n (%)	133/140 (95.0)	402/413 (97.3)	0.2	55/55 (100)	375/381 (98.4)	0.3	78/85 (91.8)	27/32 (84.4)	0.2

APACHE = acute physiology and health evaluation; IIT = intensive insulin therapy; IQR = interquartile range; MICU = medical intensive car unit; n = number; SD = standard deviation.

The studied sample comprised of a subset of long-stay patients as the median duration to the time of electrophysiological diagnosis was 18 (12 to 28) days before and 21 (13 to 32) days after implementation of IIT. As expected, both groups differed in multiple baseline characteristics such as proportion of medical patients, diagnostic group on admission, acute physiology and health evaluation (APACHE) II score and on admission blood glucose. Also exposure to known risk factors for CIP/CIM during ICU stay (Table 2) was different before and after IIT, such as treatment with noradrenaline, aminoglycosides, glucocorticoids and neuromuscular blocking agents. This necessitated MVLR analysis to correct for these imbalances, which were due to greater percentage of MICU patients in the 'before' than in the 'after' sample.

**Table 2:** incidence known risk factors for CIP/CIM, occurring during ICU stay

	**Total population n = 620**			**Surgical ICU n = 476**			**Medical ICU n = 144**		
			
	Before IIT n = 168	After IIT n = 452	p- value	Before IIT n = 62	After IIT n = 414	p-value	Before IIT n = 106	After IIT n = 38	p-value
Treatment with noradrenaline									
Treated patients, total n (%)	84/142 (59.2)	345/399 (86.5)	**< 0.0001**	37/53 (69.8)	319/366 (87.2)	**< 0.0001**	47/89 (52.8)	26/33 (78.8)	**0.007**
Number of days treatment, median (IQR)	2(0 to 9)	8 (3 to 16)	**< 0.0001**	6(0 to 15)	9(4 to 17)	**0.03**	1(0 to 6)	9(4 to 17)	**0.03**
Treatment with aminoglycosides									
Treated patients, total n (%)	45/142 (31.7)	82/399 (20.6)	**0.007**	14/53 (26.4)	76/366 (20.8)	0.3	31/89 (34.8)	6/33 (18.2)	0.08
Number of days treatment, median (IQR)	0 (0 to 1)	0 (0 to 0)	0.08	0 (0 to 1)	0 (0 to 0)	0.5	0 (0 to 1)	0 (0 to 0)	0.2
Treatment with glucocorticoids									
Treated patients, total n (%)	93/142 (65.5)	201/399 (50.4)	**0.002**	30/53 (56.6)	177/366 (48.4)	0.3	63/89 (70.8)	24/33 (72.7)	**0.04**
Number of days treatment, median (IQR)	4.5 (0 to 12)	1 (0 to 11)	**0.02**	1(0 to 12)	0(0 to 11)	0.6	6(0 to 12)	5(0 to 11)	0.6
Cumulative dose hydrocortisone equivalent mg (IQR)	945 (0 to 4350)	50 (0 to 2100)	**0.001**	300 (0 to 3009)	0 (0 to 1725)	0.3	1125 (0 to 5181)	833 (0 to 2695)	0.3
Treatment with NMBA									
Treated patients prolonged (min 3d bolus or drip) total n (%)	37/142 (26.1)	129/399 (32.3)	0.2	15/53 (28.3)	121/366 (33.1)	0.5	22/89 (24.7)	8/33 (24.2)	0.9
Number of days treatment at least 1 bolus or drip, median (IQR)	1 (0 to 3)	2 (1 to 4)	**0.006**	2 (0 to 4)	2 (1 to 4)	0.6	1 (0 to 3)	1 (0 to 2)	0.8
Dialysis,									
Yes	41/142 (28.9)	149/399 (37.3)	0.07	18/53 (34.0)	142/366 (38.8)	0.5	23/89 (25.8)	7/33 (21.2)	0.6
d, median (IQR)	0 (0 to 3)	0 (0 to 9)	0.06	0 (0 to 9)	0 (0 to 11)	0.6	0 (0 to 1)	0 (0 to 0)	0.7
Bacteraemia, yes, total n (%)	54/142 (38.0)	135/399 (33.8)	0.4	24/53 (45.3)	119/366 (32.5)	0.07	30/89 (33.7)	16/33 (48.5)	0.1
Time to diagnosis, days median (IQR)	18 (12 to 28)	21 (13 to 32)	**0.01**	21 (15 to 34)	22 (14 to 32)	0.7	15 (9 to 25)	12 (8 to 18)	0.2

CIM = critical illness myopathy; CIP = critical illness polyneuropathy; IIT = intensive insulin therapy; IQR = interquartile range; ICU = intensive car unit; n = number; NMBA = neuromuscular blocking agent.

### Glycaemia control and general outcome

We noticed a significant reduction of mean morning blood glucose from 144 ± 20 mg/dl before to 107 ± 10 mg/dl after IIT had become routine daily practice (p < 0.0001; Table 3). This significant difference was present in the medical as well as in the surgical ICU. There was no significant difference in duration of ICU stay, hospital stay, mortality rates, duration of mechanical ventilation and need for prolonged mechanical ventilation in the studied sample.

**Table 3:** Outcome characteristics of the studied sample of long-stay patients

	**Total population n = 620**			**Surgical ICU n = 476**			**Medical ICU n = 144**		
			
**Outcome before and after IIT**	**Before IIT n = 168**	**After IIT n = 452**	**p- value**	**Before IIT n = 62**	**After IIT n = 414**	**p- value**	**Before IIT n = 106**	**After IIT n = 38**	**p- value**
***General outcome***									

Mean glyc mg/dl, (mean ± SD)	144 ± 20	107 ± 10	**< 0.0001**	142 ± 18	107 ± 10	**< 0.0001**	145 ± 21	111 ± 15	**< 0.0001**
ICU stay, days, median (IQR)	37 (22 to 54)	41 (25 to 61)	0.07	45 (27 to 77)	41 (27 to 61)	0.4	32 (20 to 50)	24 (16 to 52)	0.3
Hospital stay, days, median (IQR)	61 (33 to 106)	60 (42 to 98)	0.4	74 (38 to 130)	61 (43 to 100)	0.3	50 (32 to 95)	47 (27 to 78)	0.4
Hospital mortality, total n (%)	66/152 (43.4)	170/425 (40.0)	0.5	23/54 (42.6)	154/389 (39.6)	0.7	43/98 (43.9)	16/36 (44.4)	0.9
Mechanical ventilation ≥ 14 days, total n (%)	84/142 (59.2)	259/399 (64.9)	0.2	38/53 (71.7)	248/366 (67.8)	0.6	46/89 (51.7)	11/33 (33.3)	0.07
									
***Electrophysiological data***									

Spontaneous electrical activity present, total n (%)	125/168 (74.4)	220/452 (48.7)	**< 0.0001**	49/62 (79.0)	209/414 (50.5)	**< 0.0001**	76/106 (71.7)	11/38 (28.9)	**< 0.0001**
SNAP UL									
absolute value (uV), median (IQR)	6 (0 to 10)	8 (4–13)	**0.0002**	6 (3–9)	8 (4–13)	**0.02**	6 (0–10)	6 (4–13)	0.08
percentage of normal median (IQR)	75 (0 to 125)	100 (50 to 162)	**0.0002**	75 (34 to 113)	100 (50 to 163)	**0.02**	75 (0 to 125)	80 (50 to 163)	0.08
SNAP LL									
absolute value (uV), median (IQR)	4 (0 to 8)	5 (0 to 8)	0.3	5 (0 to 8)	5 (0 to 8)	0.3	2 (0 to 6)	5 (0 to 8)	0.1
percentage of normal median (IQR)	83 (0 to 200)	100 (0 to 200)	0.5	133 (0 to 250)	100 (0 to 200)	0.09	27 (0 to 163)	102 (0 to 197)	0.1
Voluntary motor unit potential recruitment obtained, total n (%)	90/168 (53.6)	247/452 (54.6)	0.8	36/62 (58.1)	221/414 (53.4)	0.5	54/106 (50.9)	26/38 (68.4)	0.06
myogenic component present, total n (% of all patients)	27/168 (16.1)	45/452 (10.0)	**0.04**	12/62 (19.4)	39/414 (9.4)	**0.02**	15/106 (14.1)	6/38 (15.8)	0.8
myogenic component, total n (% of patients in whom contraction achieved)	27/90 (30.0)	45/202 (18.2)	**0.02**	12/36 (33.3)	39/221 (17.6)	**0.03**	15/54 (27.8)	6/26 (23.1)	0.6

IIT = intensive insulin therapy; IQR = interquartile range; LL = lower limbs; SD = standard deviation; SNAP = sensory nerve action potential; UL = upper limbs.

### Electrophysiological data

We found the incidence of CIP/CIM as defined above in the patients who were electrophysiologically evaluated, to be significantly reduced from 125/168 (74.4%) to 220/452 (48.7%) after IIT (p < 0.0001). This reduction was present among MICU patients (76/106 (71.7%) to 11/38 (28.9%), p < 0.0001) as well as SICU patients (49/62 (79.0%) to 209/414 (50.5%), p < 0.0001). After correction for baseline risk factors and risk factors occurring during ICU stay (Table 4), MVLR analysis showed that the implementation of IIT was indeed an independent protective factor for the occurrence of CIP/CIM (odds ratio (OR) 0.25 (95% confidence interval (CI): 0.14 to 0.43), p < 0.0001; Table 5). Furthermore, in the upper limbs, absolute and relative values of SNAPs were significantly improved after IIT (p = 0.002). In the lower limbs, the average SNAP was about 1 μV higher in the IIT group, but this difference was not significance.

**Table 4:** Univariate analysis of risk factors for CIP/CIM and prolonged mechanical ventilation

	**CIP/CIM Total population, n = 620**			**Prolonged mechanical ventilation Total population, n = 541**		
		
	CIP/CIM n = 345	No CIP/CIM n = 275	p- value	Prolonged mechanical ventilation	No prolonged mechanical ventilation	p- value
***Therapy***						

IIT total n (%)	220/345 (63.8)	232/275 (84.4)	**< 0.0001**	259/343 (75.5)	140/198 (70.7)	0.2
***Baseline***						

Male/female sex, n (%)	239/345 (69.3)	171/275 (62.2)	0.06	242/343 (70.6)	118/198 (59.6)	**0.009**
Age, years (mean ± SD)	62 ± 14	63 ± 15	0.4	62 ± 14	64 ± 15	0.2
ICU type (MICU, %)	87/345 (25.2)	57/275 (20.7)	0.2	57/343 (16.6)	65/198 (32.8)	**< 0.0001**
Baseline APACHE II, median (IQR)	15 (11 to 22)	15 (11 to 22)	0.4	15 (12 to 22)	16 (11 to 23)	0.7
On admission blood glucose, mg/dl median (IQR)	137 (109 to 174)	139 (112 to 181)	0.5	139 (111 to 175)	139 (113 to 183)	0.6
On admission mechanical ventilation, total n (%)	298/306 (97.4)	237/247 (96.0)	0.3	332/341 (97.4)	185/194 (95.4)	0.2
Diagnostic group, total n (%) of the category			0.3			0.4
						
Abdominal/gastrointestinal/liver	39/71 (54.9)	32/71 (45.1)		45/67 (67.2)	22/67 (32.8)	
Cardiovascular	91/171 (53.2)	80/171 (46.8)		111/165(67.3)	54/165 (32.7)	
Cerebral/neurological	26/60 (43.3)	34/60 (56.7)		34/53 (64.2)	19/53 (35.8)	
Haematological/oncologic/transplant	15/31 (48.4)	16/31 (51.6)		19/27 (70.4)	8/27 (29.6)	
Other	42/73 (57.5)	31/173 (42.5)		38/70 (54.3)	32/70 (45.7)	
Polytrauma	221/37 (59.5)	15/37 (40.5)		22/32 (68.8)	10/32 (31.2)	
Respiratory/thoracic	85/136 (62.5)	51/136 (37.5)		74/127 (58.3)	53/127 (41.7)	
History of diabetes, total n (%)			0.2			0.7
Insulin treated	21/317 (6.6)	16/254 (6.3)		25/343 (7.3)	11/198 (5.6)	
Oral antidiabetic treatment and/or diet	22/317 (6.9)	20/254 (7.9)		24/343 (7.0)	15/198 (7.6)	
***Known risk factors***						

Treatment with noradrenaline						
Treated patients, total n (%)	232/301 (77.1)	197/240 (82.1)	0.2	289/343 (84.3)	129/198 (65.2)	**< 0.0001**
Number of days treatment, median (IQR)	7(1 to 15)	6(2 to 13)	0.4	8 (3 to 13)	3 (0 to 6)	**< 0.0001**
Treatment with aminoglycosides						
Treated patients, total n (%)	76/301 (25.2)	51/240 (21.3)	0.3	77/343 (22.4)	29/198 (14.6)	**0.03**
Number of days treatment, median (IQR)	0 (0 to 1)	0 (0 to 0)	0.5	0 (0 to 0)	0 (0 to 0)	0.1
Treatment with glucocorticoids						
Treated patients, total n (%)	165/301 (54.8)	129/240(53.8)	0.8	163/343 (47.5)	100/198 (50.5)	0.5
Number of days treatment, median (IQR)	1 (0 to 12)	1 (0 to 10)	0.4	0 (0 to 8)	1 (0 to 7)	0.9
Cumulative dose up to time t	250 (0 to 2500)	300(0 to 2500)	0.8	0 (0 to 1398)	31 (0 to 1140)	0.9
						
Treatment with NMBA						
Number of days treatment (≥ 1 bolus or drip), median (IQR)	2 (1 to 4)	1 (0 to 4)	0.06	2 (1 to 3)	1 (0 to 2)	**< 0.0001**
Patients treated prolonged (≥ 3d bolus or drip) total n (%)	95/301 (31.6)	71/240(29.6)	0.6	262/343 (76.4))	107/198 (54.0)	**< 0.0001**
Dialysis						
yes	118/301 (39.2)	72/240 (30.0)	**0.03**	126/343 (36.7)	37/198 (18.7)	**< 0.0001**
n days, median (IQR)	0 (0 to 10)	0 (0 to 5)	**0.05**	0 (0 to 6)	0 (0 to 0)	**0.0001**
Bacteraemia, yes, total n (%)	113/301 (37.5)	76/240 (31.7)	0.2	92/343 (26.8)	32/198 (16.2)	**0.004**
Time to diagnosis, d median (IQR)	22 (14 to 33)	18 (11 to 27)	**0.000 7**	-	-	-
Diagnosis of CIP/CIM during ICU stay, total n (%)	-	-	**-**	207/343 (60.4)	94/198 (47.5)	**0.004**

APACHE = acute physiology and health evaluation; CIM = critical illness myopathy; CIP = critical illness polyneuropathy; IIT = intensive insulin therapy; IQR = interquartile range; MICU = medical intensive care unit; NMBA = neuromuscular blocking agent; SD = standard deviation.

**Table 5:** Multivariate logistic regression analysis for the risk for development of CIP/CIM and prolonged mechanical ventilation

	**Risk for development of CIP/CIM^a^**		**Risk for prolonged mechanical ventilation^b^**	
		
	**OR (95% CI)**	**p-value**	**OR (95% CI)**	**p-value**
***A. Uncorrected.***				

Glycaemic control, IIT	0.33 (0.22 to 0.48)	**< 0.0001**	1.28 (0.86 to 1.89)	0.2
				
***B. Corrected for baseline risk factors.***				

Glycaemic control, IIT	0.24 (0.14 to 0.42)	**< 0.0001**	0.56 (0.32 to 0.96)	**0.04**
ICU type, medical	0.49 (0.27 to 0.90)	**0.02**	0.29 (0.16 to 0.53)	**< 0.0001**
Diagnostic category				
Cardiovascular	1.005 (0.55 to 1.82)	0.9	0.78 (0.41 to 1.47)	0.4
Cerebral/neurological	0.77 (0.37 to 1.63)	0.5	0.79 (0.36 to 1.72)	0.5
Haematological/oncological/transplant	1.04 (0.41 to 2.60)	0.9	0.96 (0.35 to 2.63)	0.9
Other	1.02 (0.50 to 20.7)	0.9	0.60 (0.29 to 1.26)	0.2
Polytrauma	1.03 (0.43 to 2.49)	0.9	0.75 (0.29 to 1.90)	0.5
Respiratory/thoracic	1.43 (0.75 to 2.71)	0.3	0.85 (0.44 to 1.64)	0.6
On admission blood glucose	0.99 (0.99 to 1.001)	0.1	0.99 (0.99 to 1.002)	0.7
Gender, female	0.68 (0.47 to 1.000)	**0.05**	0.63 (0.43 to 0.93)	**0.02**
				
***C. Corrected for baseline risk factors and known risk factors occurring during ICU stay.***				

Glycaemic control, IIT	0.25 (0.14 to 0.43)	**< 0.0001**	0.40 (0.22 to 0.72)	**0.002**
ICU type, medical	0.62 (0.33 to 1.16)	0.1	0.35 (0.18 to 0.67)	**0.002**
Diagnostic category				
Cardiovascular	0.99 (0.54 to 1.83)	0.9	0.64 (0.31 to 1.29)	0.2
Cerebral/neurological	0.83 (0.39 to 1.80)	0.6	0.87 (0.36 to 2.11)	0.8
Haematological/	1.18 (0.45 to 3.12)	0.7	0.60 (0.19 to 1.93)	0.4
oncological/transplant	1.002 (0.49 to 2.07)	0.9	0.58 (0.26 to 1.27)	0.2
Other	1.09 (0.43 to 2.73)	0.9	0.88 (0.30 to 2.57)	0.8
Polytrauma	1.38 (0.72 to 2.66)	0.3	089 (0.43 to 1.86)	0.8
Respiratory/thoracic				
On admission blood glucose	0.99 (0.99 to 1.001)	0.1	0.99 (0.99 to 1.002)	0.4
Gender, female	0.74 (0.50 to 1.09)	0.1	0.74 (0.48 to 1.12)	0.2
Number of days treatment with noradrenaline, per day added	1.002 (0.98 to 1.03)	0.8	1.16 (1.11 to 1.22)	**< 0.0001**
Cumulative dose hydrocortisone equivalent, per mg added	1.000 (1.000 to 1.000)	0.3	1.00 (1.00 to 1.00)	0.9
Treatment with aminoglycosides, yes	1.073 (0.69 to 1.68)	0.8	1.72 (1.003 to 2.96)	**0.05**
Number of days treatment with NMBAs (min 1 bolus or drip), per day added	1.04 (0.98 to 1.10)	0.2	1.15 (1.04 to 1.27)	**0.007**
Number of days treatment with dialysis, per day added	1.004 (0.98 to 1.02)	0.7	1.09 (1.03 to 1.15)	**0.004**
Time t, per day added	1.01 (0.99 to 1.03)	0.2	-	**-**
Bacteraemia, yes	-	-	2.11 (1.26 to 3.55)	**0.005**

CI = confidence interval; CIM = critical illness myopathy; CIP = critical illness polyneuropathy; ICU = intensive care unit; IIT = intensive insulin therapy; NMBA = neuromuscular blocking agent; OR = odds ratio; SD = standard deviation.

^a ^risk factors occurring during ICU stay were calculated for each patient up to the point of diagnosis of presence or absence of CIP/CIM;

^b ^risk factors occurring during ICU stay were calculated for the first 14 days.

The proportion of patients in whom voluntary contraction patterns could be obtained was not different between both patient groups (90/168 (53.6%) before and 247/452 (54.6%) after IIT, p = 0.8). However, the presence of a myopathic component in the tracings obtained, was significantly lower after IIT (27/90 (30%) versus 45/247 (18.2%), p = 0.02).

### Prolonged mechanical ventilation

In the univariate analysis, no significant reduction in the need for prolonged MV was noticed in this patient sample after instituting IIT (before: 84/142 (59.2%), after: 259/399 (64.9%), p = 0.2). MVLR, however, showed that after correction for baseline risk factors and risk factors occurring during ICU stay (Table 4), the implementation of IIT was indeed an independent protective factor for prolonged MV (OR 0.40 (95% CI: 0.22 to 0.72), p = 0.002; Table 5). Another independent protector was MICU, whereas independent risk factors were number of days treatment with noadrenaline, treatment with aminoglycosides, number of days treatment with neuromuscular blocking agents, number of days treatment with dialysis and bacteraemia. To examine the impact of the reduced incidence of CIP/CIM after IIT on the need for prolonged MV, this variable was entered into the multivariate model. This analysis showed that, first of all, CIP/CIM was an independent risk factor for prolonged MV (OR:1.61(95% CI: 1.05 to 2.45), p = 0.03), and that the beneficial effect of IIT on prolonged MV remained present after this correction (OR: 0.49 (95% CI: 0.26 to 0.92), p = 0.03).

## Discussion

This is a retrospective analysis, which was conducted to examine whether the beneficial effects of IIT on neuromuscular function of critically ill patients, as was observed in two RCTs in SICU and MICU patients, could be confirmed in routine daily practice. We therefore compared electrophysiological data and data on prolonged MV from patients screened for clinical reasons before the RCTs and after, at which moment IIT was implemented in routine daily practice. This population comprised a subset of long-stay ICU patients.

As the surgical trial was performed earlier than the medical trial, most data before implementation are derived from the MICU and most data after from the SICU. The very different patient population admitted to the MICU and SICU created a large imbalance between baseline characteristics and also known risk factors for CIP/CIM encountered during ICU stay between both groups. As shown in Tables 1 and 2, most of the imbalances are completely attributable to the different percentages of medical and surgical patients before and after IIT implementation. Strikingly, however, on admission blood glucose was significantly lower after implementation of IIT in the MICU as well as in the SICU, suggesting that in general and also outside the ICU more attention was given to glucose control. To correct for the differences in patient populations and possible changes over time in therapeutic regimens, further analyses on risk factors were corrected for all baseline characteristics and risk factors occurring during ICU stay showing at least a trend towards significance in the univariate analysis.

First of all we found that IIT in routine daily care is feasible and reduced mean morning blood glucose levels to values within the target range. As in the RCTs, we found that the incidence of CIP/CIM was markedly and to the same extent reduced after IIT became part of routine care in our critically ill patients. MVLR showed that this was indeed an independent protective effect. In this study, we diagnosed CIP/CIM solely based on the presence of abundant spontaneous electrical activity. We chose to do so first of all because compound muscle action potentials (CMAPs) and SNAPs may be aspecific in the ICU setting due to technical problems, oedema, difficult access to nerves due to wound dressings etc., whereas the presence of abnormal spontaneous electrical activity indicates without any question that a neuromuscular problem is present. In contrast to other myopathies, abnormal spontaneous electrical activity is often present in CIM. Also, by using the same definition as in the RCTs, results could be compared.

As differential diagnosis between CIP and CIM via routine NCS/EMG is often difficult because of the lack of cooperation of critically ill patients, we used the SNAPs as a surrogate marker for CIP. Although other conditions such as oedema will also influence the SNAPs, we found that these values in the upper limbs were significantly increased after implementing IIT. The absence of effect in the lower limbs is noteworthy. This may be caused by the fact that screening in the lower limbs is always performed on the sural nerve, which is vulnerable to tissue oedema. Concerning effects on myopathy, we chose to take into account only results of patients in whom voluntary contraction was possible and therefore motor unit morphology and recruitment could be assessed, because these results can reliably confirm muscle versus nerve involvement. We noticed that myopathic patterns were also significantly reduced after IIT. Mechanistically, several effects of IIT may play a role, such as improvement of the microcirculation or mitochondrial function of neurons and/or muscle cells, and an effect on the balance between anabolism and catabolism.

We found no difference in the need for prolonged MV in the overall population before and after IIT. However, after correction for baseline differences and exposure to known risk factors, implementing IIT appeared to be independently associated with reduced risk of prolonged MV. As in the RCTs, the beneficial effect of IIT on prolonged MV could not be entirely explained by the reduction in CIP/CIM. The fact that the electrophysiological diagnosis of CIP/CIM itself was an independent determinant of prolonged MV suggests that this diagnosis is indeed a clinically relevant one.

This study has some important limitations, first of all because of the retrospective nature. Because of our intention to evaluate effects of a change in glycaemic control in daily clinical practice outside the controlled setting of a study protocol, and the recent results of our two RCTs, the nature of this study inevitably was retrospective and observational. Due to the different timing of the RCTs in our SICU and MICU there was a large imbalance in characteristics between the groups before and after implementation of IIT, and some daily care practices may have changed during the study period. Although we corrected for these imbalances, some caution is needed concerning the comparability of the groups before and after implementation of IIT and the validity of MVLR to correct for this. Another approach could have been to use propensity scores. However, it was recently stated that in the great majority of published studies that have used both approaches, estimated effects from propensity score and regression methods have been similar and simulation studies further suggest comparable performance of the two approaches in many settings [16]. For this reason, and because of the practical impossibility of calculating propensity scores for patients who did not receive electrophysiological examination, this statistical method was not used in this study.

The diagnosis of CIP/CIM also had some limitations. CIP/CIM was solely diagnosed on the presence of abundant spontaneous electrical activity. Therefore, we may have missed some diagnoses because muscle membrane inexcitability was not detected. By omitting those patients with only reduced CMAPs or SNAPs and no spontaneous electrical activity, we may also have missed some early diagnoses as the reduction in amplitude of the nerve and muscle action potentials (compound sensory or motor) or both, with preserved normal conduction velocity is the first electrophysiological sign that precedes other electrophysiological signs such as fibrillation potentials and positive sharp waves [17-20]. However, based on the time to diagnosis, which was quite long (median of 22 days before and 18 days after implementation), the number of patients for whom this was the case is expected to be small. Also, although the indication for electrophysiological testing was clinical weakness and/or weaning failure, no systematic evaluation of clinical weakness was reported and more sophisticated electrophysiological testing using direct muscle stimulation could have provided more details on the effects on CIP and CIM individually.

## Conclusion

We conclude that implementing IIT into standard daily care of critically ill patients exerted a similar beneficial effect on the electrophysiological diagnosis of CIP/CIM and the need for prolonged MV, as was shown in two previous RCTs. Future research should concentrate on underlying pathophysiological mechanisms.

## Key messages

• Implementing IIT in daily care of critically ill medical and surgical patients is feasible.

• IIT reduced electrophysiological incidence of CIP/CIM in daily clinical practice in critically ill medical and surgical patients, outside the controlled setting of a study protocol.

## Abbreviations

APACHE: acute physiology and health evaluation; CI: confidence interval; CIP/CIM: critical illness polyneuropathy and/or myopathy; CMAPs: compound muscle action potentials; EMG: needle electromyography; IIT: intensive insulin therapy; MICU: medical intensive care unit; MOF: multiple organ failure; MV: mechanical ventilation; MVLR: multivariate logistic regression analysis; NCS: nerve conduction studies; OR: odds ratio; RCT: randomised controlled trial; SICU: surgical intensive care unit; SIRS: systemic inflammatory response syndrome; SNAP: sensory nerve action potential.

## Competing interests

The authors declare that they have no competing interests.

## Authors' contributions

GH analysed the data, had a major contribution to the interpretation hereof and drafted the manuscript. MS designed the study concept, collected data and had a major contribution to the interpretation hereof. PD designed the study concept, collected data and had a major contribution to the interpretation of data. NB collected data. BB collected data. WDV collected data. WR had an essential contribution to the interpretation of the data. GvdB performed the statistics, had an essential contribution to the interpretation of data and the content of the manuscript.
